# UBXN7 docks on neddylated cullin complexes using its UIM motif and causes HIF1α accumulation

**DOI:** 10.1186/1741-7007-10-36

**Published:** 2012-04-26

**Authors:** Susanne Bandau, Axel Knebel, Zoe O Gage, Nicola T Wood, Gabriela Alexandru

**Affiliations:** 1 Scottish Institute for Cell Signalling (SCILLS), College of Life Sciences, University of Dundee, Dow St, Dundee DD1 5EH, UK; 2 London School of Hygiene and Tropical Medicine, Keppel St, London WC1E 7HT, UK

**Keywords:** cullin, NEDD8, p97, ubiquitin-dependent degradation, UBXD7

## Abstract

**Background:**

The proteins from the UBA-UBX family interact with ubiquitylated proteins via their UBA domain and with p97 via their UBX domain, thereby acting as substrate-binding adaptors for the p97 ATPase. In particular, human UBXN7 (also known as UBXD7) mediates p97 interaction with the transcription factor HIF1α that is actively ubiquitylated in normoxic cells by a CUL2-based E3 ligase, CRL2. Mass spectrometry analysis of UBA-UBX protein immunoprecipitates showed that they interact with a multitude of E3 ubiquitin-ligases. Conspicuously, UBXN7 was most proficient in interacting with cullin-RING ligase subunits. We therefore set out to determine whether UBXN7 interaction with cullins was direct or mediated by its ubiquitylated targets bound to the UBA domain.

**Results:**

We show that UBXN7 interaction with cullins is independent of ubiquitin- and substrate-binding. Instead, it relies on the UIM motif in UBXN7 that directly engages the NEDD8 modification on cullins. To understand the functional consequences of UBXN7 interaction with neddylated cullins, we focused on HIF1α, a CUL2 substrate that uses UBXD7/p97 as a ubiquitin-receptor on its way to proteasome-mediated degradation. We find that UBXN7 over-expression converts CUL2 to its neddylated form and causes the accumulation of non-ubiquitylated HIF1α. Both of these effects are strictly UIM-dependent and occur only when UBXN7 contains an intact UIM motif. We also show that HIF1α carrying long ubiquitin-chains can recruit alternative ubiquitin-receptors, lacking p97's ATP-dependent segregase activity.

**Conclusions:**

Our study shows that independently of its function as a ubiquitin-binding adaptor for p97, UBXN7 directly interacts with neddylated cullins and causes the accumulation of the CUL2 substrate HIF1α. We propose that by sequestering CUL2 in its neddylated form, UBXN7 negatively regulates the ubiquitin-ligase activity of CRL2 and this might prevent recruitment of ubiquitin-receptors other than p97 to nuclear HIF1α.

## Background

Proteins destined for proteasome-mediated degradation are labeled with ubiquitin chains through the action of an enzymatic cascade consisting of a ubiquitin-activating enzyme (E1), a ubiquitin-conjugating enzyme (E2), and a ubiquitin-ligase (E3) [1]. Downstream of ubiquitylation, ubiquitin-receptors recognize the poly-ubiquitylated proteins and facilitate their degradation by the proteasome [2]. Some ubiquitin-receptors, such as PSMD4 (known as Rpn10 in yeast) and RPN13, are intrinsic to the regulatory particle of the proteasome [3,4]. Others, such as those from the RAD23 or ubiquilin families, shuttle on and off the proteasome [5]. In addition to the single subunit receptors mentioned above, a distinct class of ubiquitin-receptors endowed with ATPase activity has at its core p97 hexamers. It has been proposed that p97 functions as a 'segregase' by converting the ATP-derived energy into mechanical force [6-8]. Indeed, p97 complexes can separate their substrates from cellular structures, such as the endoplasmic-reticulum membrane [9], or from protein partners [7,10]. The p97 protein itself has little affinity for ubiquitin and relies on its interaction with ubiquitin-binding adaptors to function as a ubiquitin-receptor. Such adaptors include the NPL4/UFD1 dimer [11,12] and the UBA-UBX proteins [13]. The latter employ their UBX domain to interact with the N-terminus of p97 and the UBA (ubiquitin-associated) domain to bind ubiquitylated proteins [14]. Humans express five UBA-UBX proteins: UBXN7, FAF1, FAF2, UBXN1, and p47. A striking observation from the mass spectrometry analysis of UBA-UBX protein immunoprecipitates is their ability to interact with a large number of E3 ubiquitin-ligases [15]. These include components of cullin-RING E3 ligase (CRL) complexes and also single subunit RING- and HECT-domain E3s.

Similar to ubiquitin, the ubiquitin-like (UBL) protein NEDD8 is attached to its substrates by specific E1, E2 and E3 enzymes. To date, cullins represent the major class of proteins that are targets for neddylation [16]. The NEDD8 E3 activity in this case is provided by the dual action of DCN1 [17-19] and the RING subunit of the CRL complex [20-22]. It has been known for over a decade that cullin-neddylation is essential for the E3 activity of CRL complexes [23]. Recent structural and biochemical studies elucidated the complex molecular mechanism underlying CRL activation by the NEDD8 modification. Neddylation induces a major conformational change in the cullin that essentially allows the RING domain of RBX1 to spring free from the cullin. It is this increased flexibility of the RING-domain that ultimately translates into superior ubiquitin-ligase activity of CRLs [24,25].

**Figure 1:**
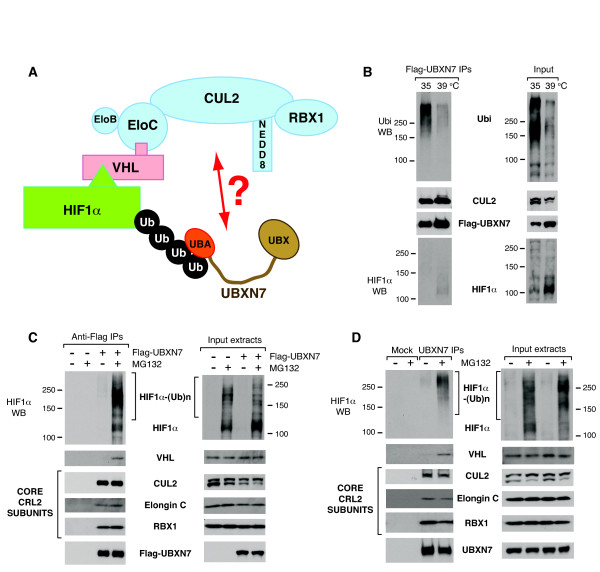
**Ubiquitin- and substrate-binding to UBXN7 do not correlate with CUL2 binding**. (**A**) Illustration of the CRL2 ubiquitin-ligase bound to its substrate HIF1α. The core subunits of CRL2 are highlighted in blue. The UBA-UBX protein UBXN7 interacts with ubiquitylated HIF1α via its UBA domain. (**B**) CUL2 interaction with UBXN7 is independent of ubiquitin-binding. Flag-UBXN7 was immunoprecipitated from cells thermo-sensitive for the ubiquitin-E1. When these cells were grown at the restrictive temperature for 20 hours, there was a marked reduction in protein ubiquitylation (right panel). CUL2-binding to UBXN7 was not affected by the reduced ubiquitin-binding observed under these conditions (left panel). (**C**) Flag-UBXN7 stably interacts with the core subunits of the CRL2 complex. Flag-UBXN7 was immunoprecipitated from HeLa cells treated or not with 10 μM MG132 for two hours. Flag-UBXN7 stably interacted with CUL2, elongin C and RBX1. The interaction with HIF1α and VHL could only be detected upon proteasome inhibition (left panel). (**D**) Endogenous UBXN7 stably interacts with the core subunits of the CRL2 complex. As in (C), but endogenous UBXN7 was immunoprecipitated from HeLa cells using specific antibodies crosslinked to protein A-beads. (**B**-**D**) The indicated proteins were detected using specific antibodies in the immunoprecipitates (left) and the input cell extracts (right).

We have previously identified HIF1α as a novel substrate of p97, with the UBA-UBX protein UBXN7 serving as the substrate-binding adaptor [15]. HIF1α heterodimerizes with HIF1β to form the HIF1 transcription factor that is essential during hypoxia for triggering the expression of specific proteins required to counteract the hypoxic stress [26]. HIF1α is continuously expressed during normoxia and is actively targeted for ubiquitin-mediated degradation through the action of a CUL2-based CRL complex (CRL2). Within CRL2, CUL2 acts as a scaffold to which the RING subunit (RBX1) and the elongin B/elongin C dimer bind. VHL docks on elongin C and directly interacts with HIF1α [27], acting as a substrate-binding adaptor for the CRL2 complex (Figure 1A). Consistent with p97 having a positive role in HIF1α degradation, HIF1α accumulates upon p97 depletion by siRNA. Paradoxically, UBXN7 depletion results in reduced levels of HIF1α, indicating that UBXN7 involvement in HIF1α degradation is more complex than anticipated [15].

Here we show that UBXN7 interaction with cullins is not mediated by its ubiquitylated substrates, but involves the direct docking of the UIM motif in UBXN7 onto the neddylated cullins. UBXN7 over-expression causes non-ubiquitylated HIF1α to accumulate in a manner that is dependent on an intact UIM motif in UBXD7. Our data suggest that UBXN7 may act as a negative regulator of CRL2 and this would favor subsequent recruitment of p97.

## Results

### Active ubiquitylation is not necessary for UBXN7 interaction with CUL2

Among human UBA-UBX proteins, UBXN7 is the most proficient in interacting with CRL subunits. Indeed, its ability to interact with CUL2 greatly outshines other UBA-UBX proteins [15]. We therefore set out to further explore UBXN7 interaction with CRL2. Our initial assumption was that the interaction of UBA-UBX proteins with E3 ubiquitin-ligases was indirect, mediated by their ubiquitylated substrates. To test this hypothesis we used the A31N-ts20 cells, which are mouse embryo fibroblasts thermosensitive for the ubiquitin-E1 [28]. When these cells are grown at the non-permissive temperature, the initial step in the protein-ubiquitylation cascade is blocked leading to a dramatic reduction in the levels of ubiquitylated proteins compared to control cells grown at 35°C (Figure 1B). As an added proof that the ubiquitylation pathway was defective, we observed HIF1α accumulation in the cells grown at 39°C. Although ubiquitin-binding to Flag-UBXN7 was drastically reduced, UBXN7 interaction with CUL2 was unaffected (Figure 1B).

### UBXN7 stably interacts with the core CRL2 complex

Next we examined UBXN7 interaction with other components of the CRL2 complex. Flag-UBXN7 effectively co-immunoprecipitated CUL2, elongin C and RBX1, which constitute the core CRL2 complex. In contrast, UBXN7 interaction with VHL and HIF1α could only be observed upon brief inhibition of the proteasome with MG132 (Figure 1C). Similar results were obtained when endogenous UBXN7 was immunoprecipitated using specific antibodies (Figure 1D).

The data presented thus far indicated that UBXN7 binding to CUL2 was not mediated by its interaction with ubiquitylated-proteins that are CRL2 substrates. This raised the interesting hypothesis that UBXN7 might interact directly with the CRL2 core complex, irrespective of whether it is charged with a substrate (Figure 1A).

### Cullin-neddylation is required for the interaction with UBXN7

**Figure 2:**
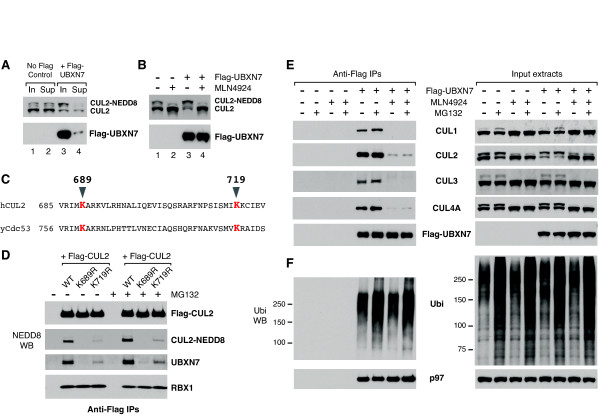
**UBXN7 exclusively interacts with neddylated cullins in cell extracts**. (**A**) UBXN7 preferentially interacts with slower-migrating, neddylated CUL2. Input cell extracts and the supernatants after Flag-UBXN7 immunoprecipitation were compared. (**B**) Flag-UBXN7 over-expression causes an up-shift of CUL2 to its neddylated form. This effect was abolished when the cells were grown in the presence of the NEDD8-E1 inhibitor MLN4924 for two hours. (**C**) Alignment of human CUL2 with the yeast cullin Cdc53. The conserved neddylation site (K689) and a more C-terminal Lys residue involved in the interaction with Dcn1 (K719) are highlighted in red. (**D**) Neddylation-defective CUL2 variants are similarly defective in interacting with endogenous UBXN7. Wild-type or mutant Flag-CUL2 was immunoprecipitated from HeLa cells treated or not with 10 μM MG132 for two hours. (**E**) MLN4924 treatment abolishes UBXN7 interaction with several endogenous cullins. (**F**) MLN4924 treatment has no effect on UBXN7 interaction with ubiquitylated-proteins or with p97. (**E**, **F**) Flag-UBXN7 was immunoprecipitated from HeLa cells treated with MG132, MLN4924, or a combination of the two. The indicated proteins were detected using specific antibodies.

We observed that UBXN7 interacted preferentially with the neddylated form of CUL2, which was largely depleted from the extracts after Flag-UBXN7 immunoprecipitation (Figure 2A, compare lanes 3 and 4). We also noticed that Flag-UBXN7 over-expression causes an up-shift of CUL2 to a slower migrating form (Figure 2B). To confirm that this is indeed neddylated-CUL2, we used a chemical inhibitor of the NEDD8-E1, MLN4924 [29]. MLN4924 abolished cullin-neddylation and also the CUL2 up-shift caused by UBXN7 over-expression (Figure 2B). To investigate the possibility that cullin-neddylation is required for the interaction with UBXN7, we created two neddylation-defective CUL2 mutants, K689R and K719R. Lys689 is the site of NEDD8-conjugation in human CUL2 and mutating this residue to arginine abolishes neddylation [30]. Lys719 is a conserved residue among cullins and its equivalent in yeast Cdc53 is part of the interaction surface with Dcn1 [17] (Figure 2C). CUL2-neddylation was largely defective in the K719R mutant (Figure 2D), presumably due to its inability to interact with the DCN1 component of the NEDD8-E3. We attempted to test whether this mutant was defective in binding the human DCN1-like protein, but DCNL1 was undetectable even in wild-type Flag-CUL2 immunoprecipitates. None of the above mutations affected CUL2 interaction with RBX1 (Figure 2D). Strikingly, there was a precise correlation between CUL2-neddylation and its ability to interact with endogenous UBXN7. UBXN7 binding was completely abolished in the K689R mutant and strongly reduced in the K719R mutant (Figure 2D). Thus, neddylation was required for CUL2 interaction with UBXN7. This is a common feature among cullins, because MLN4924 treatment prevented not only UBXN7 interaction with CUL2, but also with CUL1, CUL3 and CUL4A (Figure 2E). The defect in cullin-binding caused by MLN4924 treatment could not be rescued by simultaneous treatment with the proteasome inhibitor MG132 (Figure 2E). In contrast, MLN4924 treatment did not affect UBXN7 interaction with ubiquitylated-proteins or with p97 (Figure 2F).

### The UIM motif of UBXN7 is required to engage the NEDD8 modification on cullins

**Figure 3:**
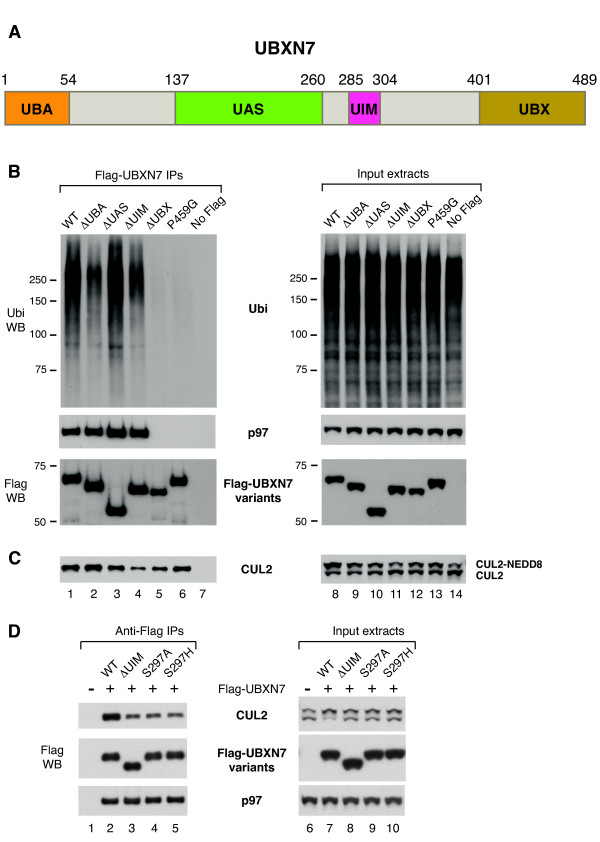
**An intact UIM motif is necessary for UBXN7 interaction with neddylated-CUL2**. (**A**) Schematic representation of human UBXN7 highlighting its various domains. (**B**-**D**) Wild-type or mutant Flag-UBXN7 was immunoprecipitated from HeLa cells. The indicated proteins were detected using specific antibodies in the immunoprecipitates (left) and the input cell extracts (right). (**B**) Either UBA- or UIM-deletion caused a reduction in ubiquitin-binding to UBXN7. UBX-deletion or a point mutation in this domain (P459G) abolished p97-binding and severely impaired the interaction with ubiquitylated proteins (left panel). (**C**) UIM-deletion, but not other mutations, caused a strong reduction in CUL2-binding to UBXN7 (left panel) and abolished the CUL2 up-shift caused by UBXN7 over-expression (right panel). (**D**) Point mutations within the UIM motif at Ser297 caused a defect in CUL2-binding to UBXN7 similar to UIM-deletion. p97-binding was not affected by these mutations (left panel).

Because UBXN7 preferentially interacted with neddylated-CUL2 and cullin-neddylation was a prerequisite for the interaction to occur, we became intrigued with the possibility that the NEDD8 modification on cullins might be directly involved in recruiting UBXN7. We therefore turned our attention to UBXN7 and the various domains that are part of its structure. At the N-terminus of UBXN7 there is a UBA domain, followed by a UAS domain of unknown function, a UIM motif and a UBX domain at the C-terminus (Figure 3A). To investigate whether any of these domains was required for UBXN7 interaction with CUL2, we compared the ubiquitin- and CUL2-binding capability of several UBXN7 variants, including wild-type, a point mutant in the UBX domain (P459G), and truncation mutants lacking either the UBA, the UAS, the UIM or the UBX domain (Figures 3B and 3C). The deletion of the UAS domain had largely no effect on UBXN7 interaction with ubiquitin, p97 or CUL2. Both ΔUBA and ΔUIM truncation mutants were partially defective in ubiquitin-binding (Figure 3B, compare lanes 2, 4 with 1). Interestingly, while ΔUBA had wild-type capability to bind CUL2 (Figure 3C,compare lanes 1 and 2), the ΔUIM truncation caused a pronounced reduction in CUL2-binding (Figure 3C, compare lanes 1 and 4). These results suggested that the UIM motif was required for CUL2-binding, while the UBA domain was not.

As expected, both UBX-deletion and a point mutation in this domain (P459G) abolished p97 binding (Figure 3B). Surprisingly, these mutations also affected ubiquitin-binding, suggesting that they have a broad effect on UBXN7 function, possibly by altering the overall structure of the protein. Although UBX mutants were severely defective in p97- and ubiquitin-binding, they largely retained their ability to interact with CUL2 (Figure 3C), supporting the notion that UBXN7 binding to CUL2 is independent of its binding to p97 or ubiquitylated-proteins.

Consistent with the reduced binding of UBXN7 ΔUIM to CUL2, over-expression of this mutant failed to cause an up-shift of CUL2 to the neddylated form (Figure 3C, compare lanes 8 and 11). In effect, CUL2 migration in cells expressing UBXN7 ΔUIM was similar to untransfected cells (Figure 3C, lanes 11 and 14).

Various residues within the UIM motif are essential for its interaction with ubiquitin [31,32]. We found that mutating Ser297 to either Ala or His causes a defect in binding neddylated-CUL2 similar to UIM deletion (Figure 3D, compare lanes 4, 5 with 3). We therefore conclude that both UIM-deletion and point mutations in this motif negatively affect UBXN7 interaction with neddylated-cullin.

**Figure 4:**
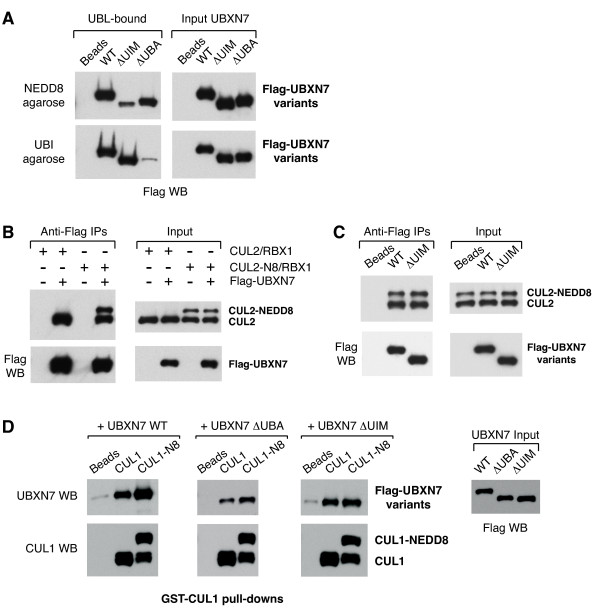
**UBXN7 directly interacts with NEDD8 and cullins *in vitro***. (**A**) The UIM motif of UBXN7 directly recognizes NEDD8. The deletion of the UIM motif exclusively reduces UBXN7 binding to NEDD8 while deletion of the UBA domain abolishes the interaction with ubiquitin. NEDD8 or ubiquitin-agarose beads were incubated with the indicated recombinant variants of UBXN7. (**B**) Wild-type UBXN7 efficiently pulls-down CUL2 irrespective of its modification status (left panel). Bacterially expressed Flag-UBXN7 was pre-incubated with full-length CUL2 either unmodified or partially neddylated and then immunoprecipitated using anti-Flag beads. (**C**) The *in vitro *interaction of UBXN7 with full-length CUL2 is not affected by UIM deletion. Wild-type or UIM-deleted Flag-UBXN7 was incubated with a mixture of neddylated and non-neddylated CUL2 and then immunoprecipitated as in (B). (**D**) Cullin-neddylation increases the binding of full-length and UBA-deleted UBXN7, but not of the UIM-deleted mutant. GSH-beads coated with either non-neddylated or a mixture of neddylated and non-neddylated CUL1(342-776)/GST-RBX1 were incubated with the indicated UBXN7 variants. Naked GSH-beads were used as a control. The Flag western blot in the right panel shows similar input levels for the three UBXN7 variants.

To further substantiate the ability of the UIM motif in UBXN7 to interact with NEDD8 rather than ubiquitin we performed *in vitro *binding assays to NEDD8- or ubiquitin-agarose. Wild-type UBXN7 was pulled-down efficiently with both types of beads (Figure 4A). Deletion of the UIM motif caused a marked reduction in NEDD8-binding and had no effect on ubiquitin-binding, while UBA-deletion abolished ubiquitin-binding and caused some reduction in NEDD8-binding as well. These data strongly support the notion that the UIM motif of UBXN7 is specialized in recognizing NEDD8 and can directly engage the NEDD8 modification on cullins.

### UBXN7 interacts with cullin-RING complexes *in vitro*

To check whether UBXN7 could interact with cullin complexes *in vitro*, we used anti-Flag beads to immunoprecipitate Flag-tagged UBXN7 incubated with either unmodified CUL2 or CUL2 that was *in vitro *neddylated. Only a fraction of CUL2 became neddylated, because RBX1 was present in substoichiometric amounts in our CUL2 preparation. We found that wild-type UBXN7 could interact efficiently with CUL2 irrespective of its neddylation status (Figure 4B). A UBXN7 variant lacking the UIM motif was equally proficient in interacting with both forms of CUL2 (Figure 4C). Under these conditions UBXN7 interaction with CUL2 does not appear to depend strictly on either UIM or NEDD8. To eliminate potential binding sites in the N-terminal half of the cullin, we then used bacterially expressed GST-RBX1 in complex with the C-terminal fragment of CUL1 (amino acids 324-776) [33]. The CUL1/RBX1 complex immobilized on glutathione-beads was either neddylated or exposed to a mock neddylation-mix without NEDD8. All UBXN7 variants tested interacted to some degree with the non-neddylated CUL1 fragment (Figure 4D). Upon CUL1-neddylation, the interaction with wild-type UBXN7 and also with the ΔUBA mutant was enhanced, while the interaction with the ΔUIM mutant remained unaffected (Figure 4D). We therefore established that UBXN7 can directly interact with cullins *in vitro *and confirmed that the UIM-NEDD8 contact has a contribution, albeit not as important as observed using cell extracts.

### UBXN7 over-expression causes HIF1α accumulation in a UIM-dependent manner

**Figure 5:**
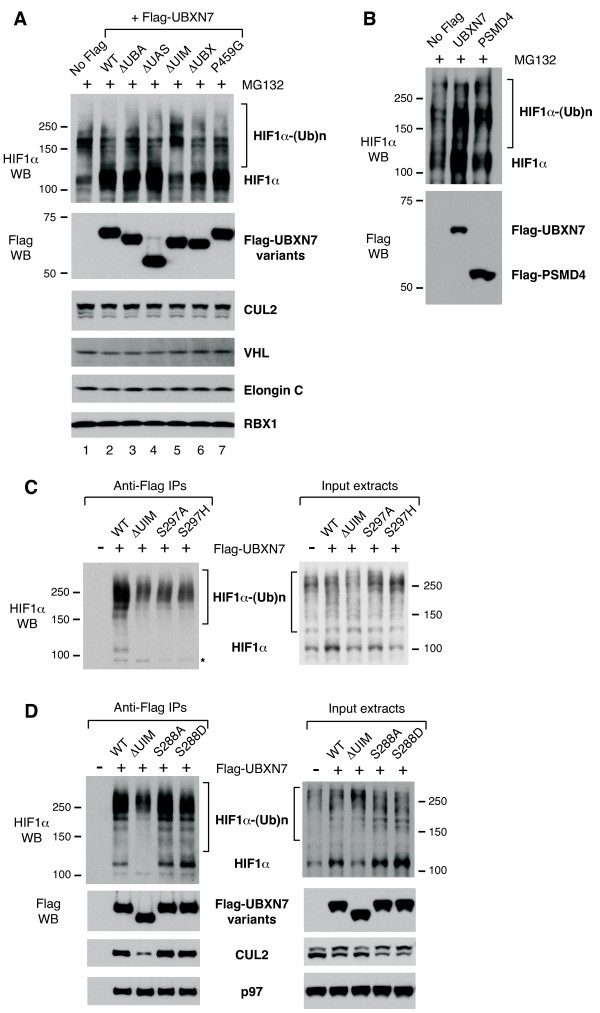
**UBXN7 over-expression causes HIF1α accumulation in a UIM-dependent manner**. (**A**) Non-ubiquitylated HIF1α accumulates in cells over-expressing wild-type Flag-UBXN7, but not in cells expressing a UIM-deleted version. 10 μM MG132 was added two hours prior to cell lysis to facilitate HIF1α detection. UBXN7 over-expression had no effect on CRL2 subunit levels. (**B**) Over-expression of another UIM-containing protein, PSMD4, does not alter HIF1α levels. (**C, D**) UIM-dependent accumulation of non-ubiquitylated HIF1α upon UBXN7 over-expression is also observed in the absence of proteasome inhibition (right panels). Wild-type UBXN7 interacted with HIF1α at various degrees of ubiquitylation, while UIM-defective UBXN7 (upon UIM deletion or point mutation at Ser297) only interacted with slow-migrating, poly-ubiquitylated HIF1α (left panels). The corresponding Flag, CUL2 and p97 western blots for the experiment in (C) are shown in figure 3D. (**D**) UBXN7 mutated at Ser288 within the UIM motif binds CUL2 (left panel) and causes HIF1α accumulation (right panel), similar to the wild-type protein. The indicated proteins were detected using specific antibodies.

Because various UBXN7 mutants had altered ability to interact with ubiquitylated-proteins or with CUL2, we checked whether their expression in the cell might have any consequence on the levels of HIF1α, which is a CRL2 substrate [27] and also interacts with UBXN7 [15]. Over-expression of wild-type UBXN7 caused a significant accumulation of HIF1α in its non-ubiquitylated form (Figure 5A, compare lanes 1 and 2). Most importantly, this effect was dependent on the UIM motif, as HIF1α levels in cells over-expressing a UIM-deleted version of UBXN7 were similar to untransfected cells (Figure 5A, lanes 1 and 5). In contrast, the levels of the CRL2 subunits CUL2, VHL, elongin C and RBX1 remained unaffected (Figure 5A). UBXN7 is unique in its ability to cause UIM-dependent accumulation of HIF1α, as over-expression of another UIM-containing ubiquitin-receptor, the proteasome subunit PSMD4, had no effect on HIF1α levels (Figure 5B).

To facilitate HIF1α detection, for the experiments shown in Figures 5A and 5B, the cells were subjected to brief inhibition of the proteasome activity prior to cell lysis. However, UIM-dependent accumulation of HIF1α upon UBXN7 over-expression could also be observed in the absence of MG132 treatment (Figures 5C and 5D, right panels). Point mutations at Ser297 in the UIM motif that negatively affect CUL2-binding (Figure 3D) also abolished HIF1α accumulation, similar to UIM-deletion (Figure 5C, right panel).

Previous reports identified a phosphorylation site within the UIM motif of human UBXN7 at Ser288 [34,35]. We therefore created a phosphorylation-defective (S288A) and a phosphorylation-mimicking mutant (S288D) of that residue to verify whether phosphorylation of Ser288 might regulate UIM function. Both mutants behaved similar to wild-type with respect to CUL2- and HIF1α-binding (Figure 5D). They also caused HIF1α accumulation, much like wild-type UBXN7 (Figure 5D, right panel). Therefore, phosphorylation at Ser288 does not appear to be critical for UIM function.

Interestingly, the defect in CUL2-binding observed for the UIM-defective mutants correlated with a complete loss of binding to non- or oligo-ubiquitylated HIF1α (Figure 5C). Nonetheless, these mutants retained the ability to interact with poly-ubiquitylated HIF1α (that is, the slowest migrating forms of HIF1α), presumably via the UBA domain.

### Long ubiquitin-chains on HIF1α cause reduced ubiquitin-receptor selectivity

**Figure 6:**
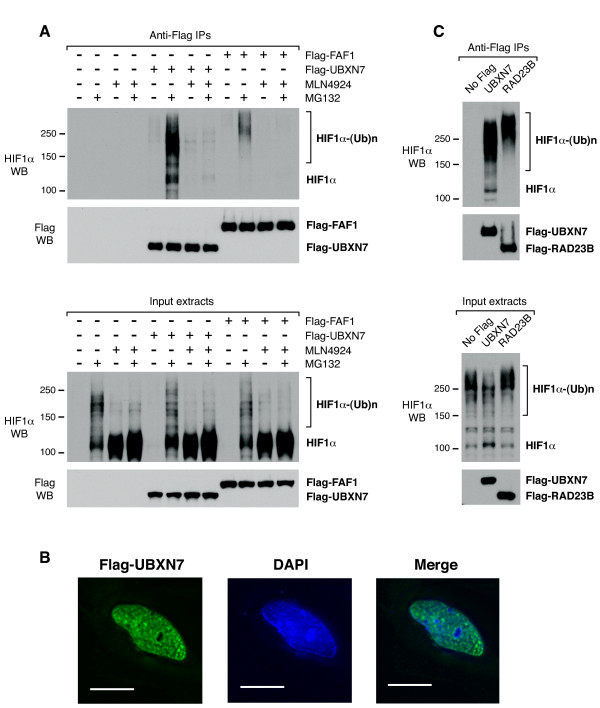
**HIF1α carrying long ubiquitin-chains becomes promiscuous in its interaction with ubiquitin-receptors**. (**A**) FAF1 interacts, albeit inefficiently, with HIF1α carrying long ubiquitin-chains (top panel). Flag-UBXN7 or Flag-FAF1 was immunoprecipitated from HeLa cells treated with 10 μM MG132, 1 μM MLN4924, or a combination of the two for two hours. (**B**) Flag-UBXN7 (green) localizes to the nucleus of HeLa cells. The DNA was stained with DAPI (blue). The scale bar represents 15 μm. (**C**) RAD23B interacts with slower-migrating, poly-ubiquitylated HIF1α (top panel). Flag-UBXN7 or Flag-RAD23B was immunoprecipitated from HeLa cells using anti-Flag beads. The indicated proteins were detected by western blotting using specific antibodies.

We previously observed that HIF1α carrying long ubiquitin-chains can interact, albeit inefficiently, with UBA-UBX proteins other than UBXN7 [15]. Consistent with it being pre-docked on the CRL2 complex, UBXN7 interacted with HIF1α at various degrees of ubiquitylation, from non- or oligo-ubiquitylated to poly-ubiquitylated (Figure 6A). In contrast, another UBA-UAS-UBX protein, FAF1, only interacted with slower migrating HIF1α (Figure 6A), similar to UBXN7 lacking the UIM motif (Figure 5D).

Flag-UBXN7 was found exclusively in the nuclei of HeLa cells (Figure 6B). RAD23B, another UBA-domain ubiquitin-receptor also localizes to the nuclear compartment [36,37] and this prompted us to check whether it may also interact with HIF1α. Like FAF1, RAD23B was able to co-immunoprecipitate HIF1α carrying longer ubiquitin-chains (Figure 6C). Hence, as the ubiquitin-chains get longer, the substrate appears less selective in its interaction with ubiquitin-receptors. Our observations suggest that UBXN7 targets mainly the nuclear pool of HIF1α and it may compete with RAD23B for ubiquitylated-HIF1α in the nucleus.

## Discussion

### UBXN7 interaction with cullins requires neddylation and is independent of the ubiquitylated substrate

Multiple lines of evidence indicate that ubiquitin/substrate-binding and cullin-binding to UBXN7 are two independent events: (1) inhibition of the ubiquitin-E1 strongly reduces ubiquitin-binding, but it has no effect on CUL2-binding to UBXN7; (2) UBXN7 interaction with HIF1α/VHL is transient and strongly enhanced upon proteasome inhibition while UBXN7 interaction with the core CRL2 complex is stable; (3) UBA-domain deletion reduces ubiquitin-binding to UBXN7, but it does not affect the interaction with CUL2.

Furthermore, several observations support the notion that neddylation is necessary for UBXN7 interaction with cullins: (1) CUL2 mutants defective to varying degrees in getting neddylated are similarly defective in UBXN7 binding; (2) chemical inhibition of the NEDD8-E1 abolishes the interaction of UBXN7 with multiple cullins; (3) *in vitro *neddylation of a CUL1-fragment stimulates its interaction with bacterially-expressed UBXN7, in a UIM-dependent manner. *In vitro*, UBXN7 can interact with non-neddylated cullins suggesting that UIM-NEDD8 may not be the only link between UBXN7 and CRLs. As we used simplified CRLs containing only the cullin and RBX1, these other binding determinants might be particularly accessible, thereby relinquishing the strict requirement for neddylation that we observed for the native form of CUL2 present in cell extracts.

### UBXN7, one domain for each interaction

The UBX domain has a ubiquitin-like structure [38] and is widely used by p97 co-factors to interact with p97 N-termini [13]. Our analysis confirms that the UBX domain is the only domain of UBXN7 required for p97 binding.

Both UBA and UIM have been characterized extensively as ubiquitin-binding modules [39]. NEDD8 and ubiquitin sequences are 57% identical resulting in a very similar three-dimensional fold, termed the ubiquitin superfold. Most importantly, the hydrophobic surface of ubiquitin (formed by Leu8, Ile44, His68 and Val70) that interacts with ubiquitin-binding domains such as UBA [40] and UIM [41] is conserved in NEDD8 [42]. UBA and UIM interact with NEDD8 *in vitro *[43,44] and also with another domain from the ubiquitin superfold family, the UBL domain [45]. In principle, either UBA or UIM could serve as a docking site for neddylated-cullins.

We show that within UBXN7, UBA and UIM play distinct roles. In cell extracts, the UBXN7 mutant lacking the UBA domain is fully competent in interacting with cullins, thus ruling out the involvement of this domain in cullin-binding. This mutant is the least competent in ubiquitin-binding, but it is not fully defective. It is possible that, in this case, ubiquitin-binding is not direct, but mediated by the UIM motif bound to cullins. In contrast, UBXN7 lacking the UIM motif or carrying point mutations therein is strongly defective in cullin-binding. The residual CUL2-binding observed for the ΔUIM mutant may be mediated by its UBA-dependent interaction with ubiquitylated substrates of CRL2. Indeed this mutant retains the ability to interact with HIF1α carrying long ubiquitin-chains. We argue that the UIM motif contributes to the direct binding of UBXN7 to neddylated-cullins. This conclusion is strongly supported by our *in vitro *binding assays where a UBXN7 variant lacking the UIM motif becomes defective in binding to NEDD8- but not ubiquitin-agarose. This experiment also clarifies that the UIM motif recognizes the NEDD8 modification *per se *rather than the neddylated conformation of cullins.

**Figure 7:**
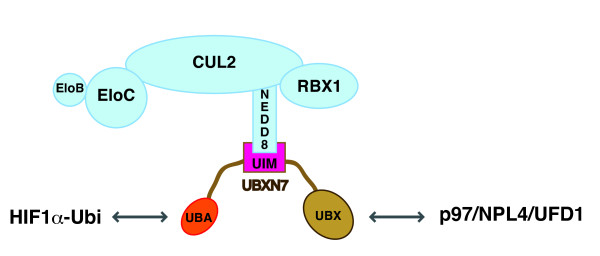
**UBXN7 docks on neddylated cullins using its UIM motif**. Each domain in UBXN7 mediates a specific interaction: the UBA domain interacts with ubiquitylated HIF1α, the UIM motif anchors the neddylated CRL complex and the UBX domain recruits the p97/NPL4/UFD1 complex.

The data presented here suggest that each of the three domains found in UBXN7 enables a specific interaction to occur. Thus, the UIM motif allows docking of UBXN7 on neddylated-cullins, the UBA domain is required for binding ubiquitylated-protein substrates, and the UBX domain recruits the p97 complex (Figure 7). Future work will reveal the role played by the UAS domain.

### Processivity versus selectivity in CRL-dependent degradation

Having established that UBXN7 can directly interact with neddylated CRL complexes, a key question is whether this interaction might have any effect on the ubiquitin-ligase activity of CRLs.

The data presented here lead us to speculate that UBXN7 is not only a ubiquitin-binding adaptor for p97, but it may also embody a novel mechanism of CRL inhibition. This would be in agreement with our previous observation that UBXN7 depletion by siRNA causes a reduction in HIF1α levels and not HIF1α accumulation as would be expected were it simply mediating HIF1α interaction with p97 [15].

Our finding that UBXN7 over-expression causes the accumulation of HIF1α mostly in its non-ubiquitylated form suggests that the UIM-NEDD8 interaction would have a negative effect on ubiquitin-chain elongation, that is, reduce the processivity of the CRL ubiquitin-ligase. UBXN7 over-expression causes not only HIF1α accumulation, but also converts CUL2 to its neddylated form. Because both effects are entirely dependent on the UIM motif, it is tempting to propose that by sequestering CUL2 in its neddylated form, UBXN7 might sterically hinder the transition of the CRL complex to an open conformation and thereby mitigate the positive effect NEDD8 has on CRL E3 activity.

We find that ubiquitin-receptor selectivity is compromised when HIF1α carries long ubiquitin-chains. Hence, we suggest that reduced CRL processivity would favor p97 recruitment to the UBX-domain of UBXN7 rather than recruitment of alternative ubiquitin-receptors to a fast-growing ubiquitin chain. Through its prior docking on the neddylated-CRL, UBXN7 would be ideally poised to modulate substrate-ubiquitylation and to shift the balance towards p97 recruitment.

### UBXN7 recruits p97 to nuclear HIF1α

Employing p97 complexes as the ubiquitin-receptor of choice is particularly important in the nucleus, where HIF1α forms complexes with HIF1β and associates with the promoters of its target genes [46-48]. Among the various ubiquitin-receptors, p97 uniquely provides the segregase activity required to release nuclear HIF1α from its protein partners and/or from chromatin prior to its degradation. Endogenous HIF1α is found in the nuclei of normoxic cells from normal and tumor tissues [49-51] and poly-ubiquitylated HIF1α is detected exclusively in the nuclei of normoxic HeLa cells [52]. Consistent with the notion that UBXN7/p97 targets specifically nuclear HIF1α, we find that Flag-UBXN7 localizes to the nucleus of normoxic HeLa cells.

## Conclusions

Herein we show that among the ubiquitin-binding adaptors of p97, UBXN7 has the unique ability to directly dock onto neddylated cullins. This function depends on the UIM motif that is only found in the UBXN7 co-factor of p97. Furthermore, UBXN7 interaction with neddylated CUL2 appears to negatively affect its ubiquitin-ligase activity as UBXN7 over-expression causes the accumulation of non-ubiquitylated HIF1α in a UIM-dependent manner.

It has been puzzling why p97 has such a multitude of ubiquitin-binding co-factors, for example, NPL4, UFD1, various UBA-UBX proteins, PLAA. Our data indicate that the role of UBXN7 as a substrate-binding adaptor for p97 is secondary to its ability to interact with CRLs and possibly to modulate their activity. UBXN7 interaction with cullins does not require p97, while UBXN7 interaction with NPL4/UFD1 is mediated by p97 [15], suggesting that UBXN7 acts upstream of NPL4/UFD1 in the p97 pathway of ubiquitin-dependent degradation. It is feasible to assume that various adaptors of p97 function in a sort of relay and their temporal succession is dictated by functions other than mere ubiquitin recognition. Future work will tell for how many of the above proteins their ubiquitin-binding capacity is just one facet of a more complex function.

## Methods

### Cloning Information

Human UBXN7 [GenBank: NM_015562] was amplified from EST IMAGE 5294894. For mammalian expression, wild-type and mutant UBXN7 variants were subcloned as BamH1/Not1 inserts into pCMV5-Flag. For bacteria expression, UBXN7 variants were subcloned into a modified pGEX6P-1 vector containing a TEV protease site and a Flag tag downstream of the GST. Human CUL2 [GenBank: NM_003591] and RAD23B [GenBank: NM_002874.3] were amplified from EST IMAGE 4104375 and 3906269, respectively, for subcloning into pCMV5-Flag as BamH1/Not1 inserts. Human FAF1 [GenBank: NM_007051.2] and PSMD4 [GenBank: NM_002810.2] were amplified from EST IMAGE 5928559 and 6285035, respectively, for subcloning into pCMV5-Flag as Sal1/Not1 inserts. To construct the baculovirus vector for dual expression of GST-CUL2 and HIS_6_-RBX1, human CUL2 was subcloned as a BamH1/Not1 insert into the P_PH _driven cassette of pFastBac-Dual-GST. RBX1 [GenBank: NM_014248.2] was amplified from EST IMAGE 3138751 adding an Nhe1 site and 6HIS tag on the 5' primer and a Kpn1 site on the 3' primer and subcloned into the P_P10 _driven cassette of pFastBac-Dual-GST-CUL2.

PCR reactions were carried out using KOD Hot Start DNA Polymerase (Merck Millipore, Darmstadt, Germany). All full-length PCR products were cloned into pSc-B (Agilent Technologies, Santa Clara, CA, USA) and fully sequenced prior to further subcloning. All mutations and deletions were made following the Quickchange method (Agilent Technologies), but using KOD Hot Start DNA Polymerase. DNA sequencing was performed by the Sequencing Service at the College of Life Sciences (CLS), University of Dundee.

### Cell Extracts and Immunoprecipitation

For immunoprecipitation experiments, the cells were lysed in buffer A (50 mM N-2-hydroxyethylpiperazine-N'-2-ethanesulfonic acid (HEPES)/KOH, pH 7.2; 5 mM Mg(OAc)_2_; 70 mM KOAc; 0.2% Triton X-100; 10% glycerol; 0.2 mM ethylenediaminetetraacetic acid (EDTA); protease inhibitors) and incubated with anti-UBXN7 antibodies crosslinked to Protein A-agarose or anti-Flag beads (Sigma, Saint Louis, MO, USA). For the experiment in Figure 5D, PhosSTOP phosphatase inhibitor (Roche. Mannheim, Germany) was also added to the lysis buffer.

Total extracts were prepared using buffer B (50 mM HEPES/KOH, pH 7.2; 400 mM NaCl; 1% NP-40; 0.2 mM EDTA; 10% glycerol; protease inhibitors) to facilitate extraction of nuclear HIF1α.

### Antibodies and Chemicals

The following antibodies were used for protein detection by western blotting: mouse anti-Flag M2 (Sigma), mouse anti-ubiquitin FK2 (Enzo, Farmingdale, NY, USA), mouse anti-p97 (Fitzgerald, North Acton, MA, USA), mouse anti-CUL3, mouse anti-elongin C (BD Transduction Laboratories, San Jose, CA, USA), rabbit anti-CUL4A (Cell Signalling, Danvers, MA, USA), rabbit and mouse anti-CUL1, rabbit anti-CUL2, rabbit anti-NEDD8 (Invitrogen, Camarillo, CA, USA), rabbit anti-RBX1 (Thermo, Fremont, CA, USA), rabbit anti-VHL (Santa Cruz Biotechnology, Santa Cruz, CA, USA), rabbit anti-HIF1α (Novus, Littleton, CO, USA), rabbit anti-UBXN7 (courtesy of Millipore, Billerica, MA, USA). MG132 (Enzo) was added at 10 μM to the tissue culture media for two hours prior to cell lysis. The Division of Signal Transduction Therapy (DSTT) at the CLS, University of Dundee synthesized MLN4924, as described previously [53]. The cells were incubated with 1 μM MLN4924 for two hours.

### Recombinant Protein Expression and Purification

The Protein Production and Assay Development Team (PPADT) at SCILLS produced the various recombinant proteins in bacteria, as follows. Expression vectors for full length, UBA- or UIM-deleted UBXN7 were transformed into BL21 DE3 cells. Overnight cultures were grown in LB medium (1% tryptone, 0.5% yeast extract, 1% NaCl) supplemented with carbenicillin. Autoinduction medium was inoculated and the cells were left to grow at 37°C until the OD_600 _reached about 1.5. The temperature was then dropped to 15°C and the cells were left for about 16 hours to express the protein. The cells were collected by centrifugation and resuspended in 50 mM Tris-HCl pH 7.5, 250 mM NaCl, 0.4% Triton X-100, 0.1 mM EDTA, 0.1 mM ethylene glycol tetraacetic acid (EGTA), 1 mM dithiothreitol (DTT) and protease inhibitors. The suspension was sonicated and the insoluble material was sedimented by centrifugation at 4°C, 28,000 g for 20 minutes. The supernatant was incubated with GSH-sepharose for one hour. The sepharose was washed four times and UBXN7 was recovered upon cleavage with TEV protease. The proteins were further purified by chromatography over a Superdex 75 column after which protein purity exceeded 90%.

The vector expressing Flag-CUL1(324-776)/GST-HA-RBX1 [33] was also transformed into BL21 cells, but grown in LB/ampicillin and induced with 0.1 mM isopropyl-beta-D-thiogalactopyranoside (IPTG) at an OD_600 _of 0.7. This was then left to express at 15°C overnight and the lysate was prepared as described above.

The dual expression vector encoding GST-CUL2/HIS_6_-RBX1 was used to generate recombinant baculoviruses using the Bac-to-Bac system (Invitrogen) following the manufacturer's protocol. These baculoviruses were used to infect *Spodoptera frugiperda *21 cells (1.5 × 10^6^/ml) at a multiplicity of infection of 5 and the infected cells were harvested 48 hours post-infection. GST-CUL2/RBX1 was purified on GSH-Sepharose and dialysed into 50 mM Tris-HCl pH 7.5, 0.1 mM EGTA, 150 mM NaCl, 270 mM sucrose, 0.03% Brij-35, 0.1% 2-mercaptoethanol, 1 mM benzamidine, 0.1 mM phenylmethylsulfonyl fluoride (PMSF).

### *In Vitro *Binding Assays

The Flag-CUL1(324-776)/GST-HA-RBX1 complex was immobilized on GSH-sepharose (GE). For each binding assay, 10 μl beads carrying approximately 1 μg CUL1/RBX1 were incubated for 30 minutes at 30°C with a neddylation reaction mix containing NEDD8 E1 (PPADT, SCILLS), NEDD8 E2 (Ubiquigent, Dundee, UK), NEDD8 and ERS (BostonBiochem, Cambridge, MA, USA) in buffer C (50 mM HEPES/KOH, pH 7.5; 60 mM KOAc; 5 mM MgCl_2_; 5% glycerol, 1 mM DTT). Mock neddylation reactions were performed in parallel by omitting NEDD8 from the mixture. The beads were then washed and incubated for one hour with 3 μg of wild-type or mutant Flag-UBXN7 in buffer C without DTT and supplemented with 0.1% Triton X-100 (buffer D). After washing the beads, the bound proteins were eluted with Laemmli buffer. The binding assays were also performed using naked beads to account for non-specific UBXN7 binding to the beads.

GST was cleaved off CUL2 with PreScission protease and the resulting CUL2/RBX1 was neddylated as above. CUL2/RBX1 (1.5 μg) was pre-incubated with 1.5 μg Flag-UBXN7 (approximately 1.5 times molar excess to CUL2) for 30 minutes, followed by one hour incubation with 10 μl anti-Flag beads in buffer D. The proteins bound to beads were eluted by boiling in Laemmli buffer.

Wild-type or mutant UBXN7 (25 μg) was incubated for one hour with 10 μl NEDD8- or ubiquitin-agarose (BostonBiochem) in buffer D and the bound proteins were eluted as above.

### Immunofluorescence Staining and Microscopy

For Flag-UBXN7 immunostaining, cells were grown on coverslips and fixed with ice-cold methanol for six minutes at -20°C. Cells were then blocked in 1% BSA/PBS for 30 minutes and subsequently incubated with mouse anti-Flag M2 (Sigma) antibodies in 3% BSA/PBS, for one hour at room temperature. After washing with PBS, cells were incubated with donkey anti-mouse FITC-conjugated antibodies (Jackson ImmunoResearch, West Grove, PA, USA) for 45 minutes. The cell nuclei were stained with 4',6-diamidino-2-phenylindole (DAPI, Invitrogen). The coverslips were mounted onto glass slides using hydromount (National Diagnostics, Atlanta, GA, USA).

Images were obtained with a DeltaVision Spectris microscope (Applied Precision), using a CoolSNAP HQ camera (Roper) and a 60 × 1.4 NA objective (Olympus). The SoftWorx software (Applied Precision) was used for acquisition and deconvolution.

## Abbreviations

ATP: adenosine triphosphate; BSA: bovine serum albumin; CLS: College of Life Sciences; CRL: cullin-RING E3 ligase; CRL2: CUL2-based CRL; DAPI: 4',6-diamidino-2-phenylindole; DSTT: Division of Signal Transduction Therapy; DTT: dithiothreitol; EDTA: ethylenediaminetetraacetic acid; EGTA: ethylene glycol tetraacetic acid; ERS: energy regeneration solution; FITC: fluorescein isothiocyanate; GSH: glutathione; GST: glutathione S-transferase; HECT: homologous to E6-AP carboxyl terminus: HEPES: N-2-hydroxyethylpiperazine-N'-2-ethanesulfonic acid; IPTG: isopropyl-beta-D-thiogalactopyranoside; KOAc: potassium acetate; KOH: potassium hydroxide; LB: Luria-Bertani media; Mg(OAc)_2_: magnesium acetate; NaCl: sodium chloride; OD: optical density; PBS: phosphate buffered saline; PCR: polymerase chain reaction; PMSF: phenylmethylsulfonyl fluoride; PPADT: Protein Production and Assay Development Team; RING: really interesting new gene; SCILLS: Scottish Institute for Cell Signalling; siRNA: small interfering RNA; TEV: Tobacco Etch virus; UAS: domain of unknown function; UBA: ubiquitin-associated domain; UBL: ubiquitin-like protein; UBX: ubiquitin regulatory X domain; UIM: ubiquitin-interacting motif.

## Competing interests

The authors declare that they have no competing interests.

## Authors' contributions

SB performed the experiments shown in Figures 1D, 3D, 5B, C, 6B and 6C. GA performed all the other experiments, conceived the study and wrote the manuscript. ZG and NW did the cloning. AK coordinated expression and purification of the proteins used for *in vitro *assays. All authors read and approved the final manuscript.
